# Cervical Spondylodiscitis in an Infant with Torticollis

**DOI:** 10.5334/jbsr.2454

**Published:** 2021-06-10

**Authors:** Brecht Van Berkel, Kristin Suetens, Luc Breysem

**Affiliations:** 1 UZ Leuven, BE

**Keywords:** Spondylodiscitis, MRI, pediatric, torticollis, cervical, CT

## Abstract

**Teaching point:** Narrowing of the intervertebral space and destruction of the adjacent vertebral end plates on conventional radiography or CT should raise suspicion for spondylodiscitis in symptomatic infants.

## Case Report

**Figure 1:**
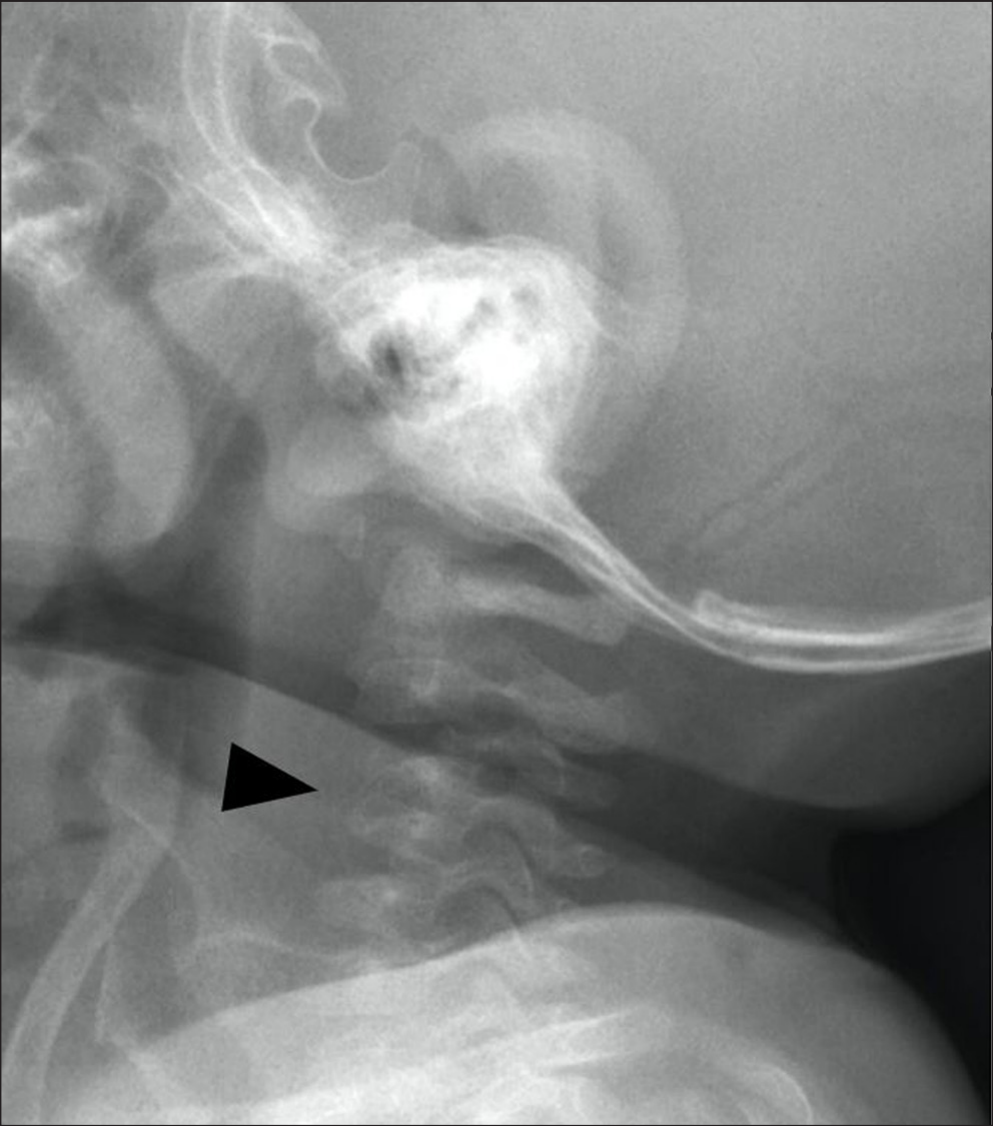


**Figure 2:**
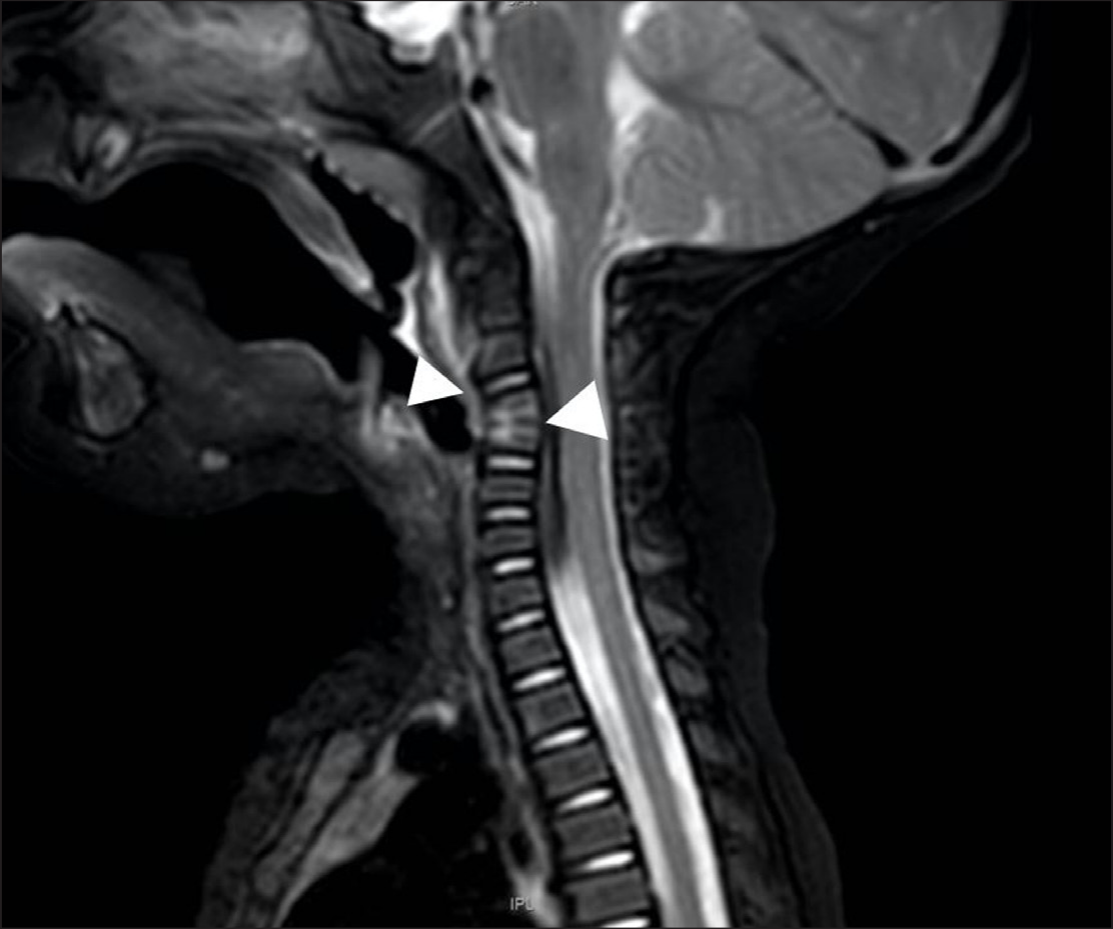


**Figure 3:**
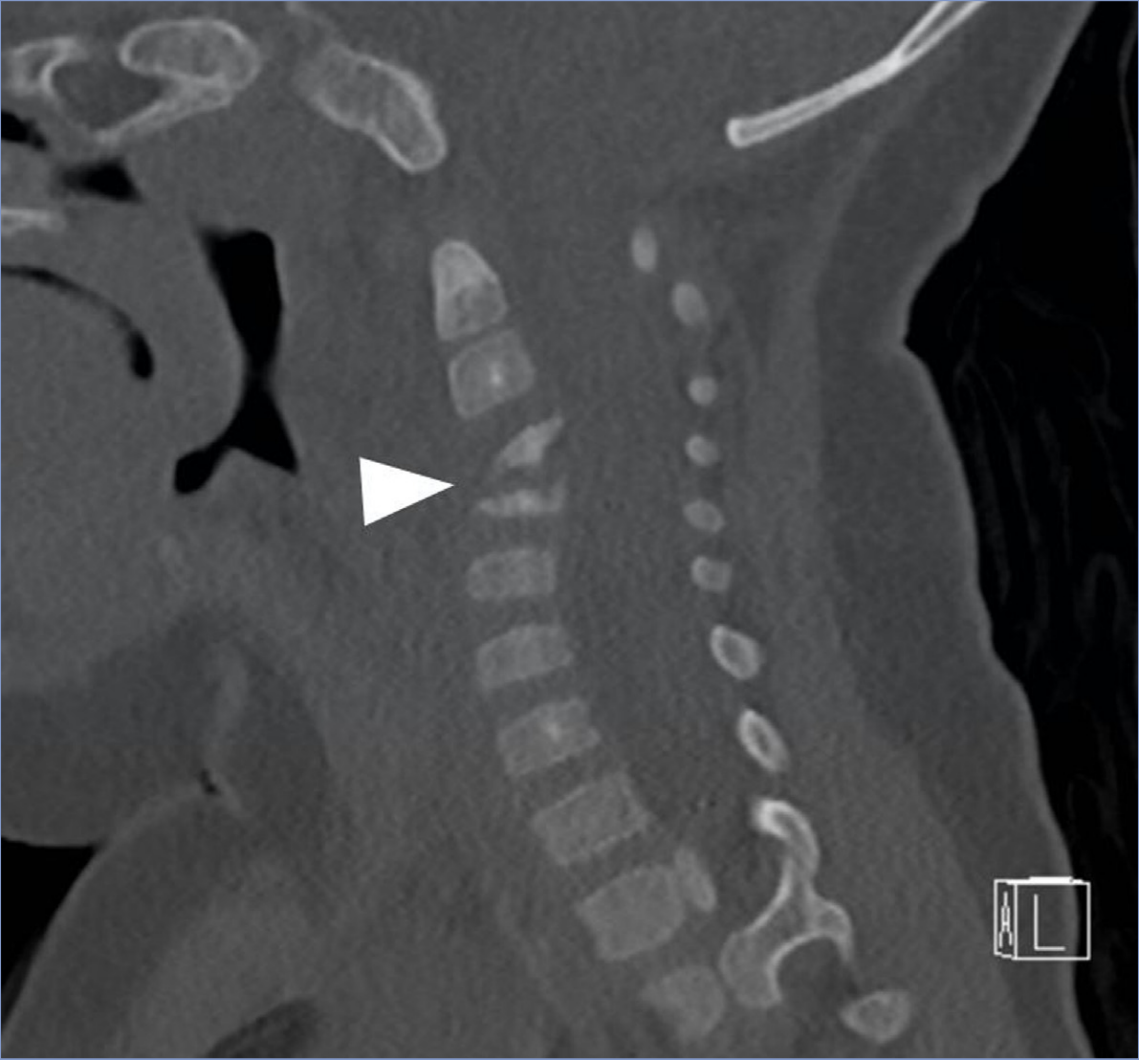


An eight-month-old infant presented at the emergency department with a history of torticollis for six weeks. Blood results showed no elevated inflammatory parameters. Vertical lateral X-ray of the cervical spine demonstrated a kyphotic angulation at the level of C3–C4, narrowing of the intervertebral disc space, irregular end plates, and loss of height of the vertebral body of C3 and C4 (arrowhead, ***Figure 1***). Magnetic resonance imaging (MRI) confirmed narrowing of the intervertebral disc space and loss of height of vertebral bodies C3 and C4 and irregular alignment of the end plates. On the sagittal T1-weighted Short-tau inversion-recovery (STIR) images, the hyperintense signal in the vertebral bodies of C3 and C4, as well as in the surrounding tissues (arrowheads) were compatible with a widespread area of bone and soft tissue oedema (***Figure 2***). There were no diffusion-restricted areas and no accompanying fluid collections. As the diagnosis of cervical spondylodiscitis was likely, intravenous antibiotics (cefazolin) were started and continued for seven days (150 mg/kg/day) until clinical improvement, which confirmed the presumptive diagnosis. To evaluate the extent of bone destruction a complementary spine computed tomography (CT) was performed two days later, confirming partial bone destruction of the C3 and C4 vertebral bodies and their intervertebral space (arrowhead, ***Figure 3***).

## Discussion

Clinical presentation of spondylodiscitis in infants is nonspecific in most cases, with symptoms such as neck or back pain. Fever is only present in <50% of patients and blood results are mostly normal; therefore, imaging is often the key to diagnosis. This was also illustrated in our case report. In general, conventional radiography should be the first-line imaging modality for paediatric patients with acquired torticollis, followed by CT and MRI. This is different in congenital torticollis, where the first-line imaging modality of choice is ultrasound.

Regarding cervical spondylodiscitis abnormal findings on plain radiography and CT are narrowing of the intervertebral space and destruction of the adjacent vertebral end plates in variable degrees and these should not be missed [1].

MRI is the most sensitive imaging modality to evaluate spondylodiscitis in children. In early stages, imaging findings are consistent with discitis, that is, T2 hyperintensity of the disc and even volume increase of the disc due to oedema. This stage is followed by a loss of the intervertebral disc height with subtle signal changes of the vertebral end plates and enhancement of the annulus fibrosus after gadolinium administration. After further disease progression, the end plates may become irregular and T2 hyperintense, as well as the rest of the vertebral bodies, with contrast enhancement of both the vertebral structures, the remaining disc and the surrounding soft tissue [1].

Regarding the differential diagnosis: lymphoma, chronic recurrent multifocal osteomyelitis (CRMO), and Langerhans cell histiocytosis have to be considered. All these entities have relatively similar imaging findings compared to cervical spondylodiscitis. In our case, the good response to antibiotics ruled out these alternative diagnoses.
